# Skin pigmentation bias in regional brain oximetry measurements?

**DOI:** 10.1186/s13054-022-04295-4

**Published:** 2023-01-10

**Authors:** V. Quaresima, F. Scholkmann, M. Ferrari

**Affiliations:** 1 Department of Life, Health and Environmental Science, University of L’Aquila, L’Aquila, Italy; 2 Biomedical Optics Research Laboratory, Department of Neonatology, University Hospital Zurich, University of Zurich, Zurich, Switzerland

To the Editor,

On November 1 2022, on the Food and Drug Administration (FDA) virtual public meeting of the Center for Devices and Radiological Health (CDRH), the Anesthesiology and Respiratory Therapy Devices Panel of the Medical Devices Advisory Committee discussed several recent studies reporting pulse oximeters to provide less accurate readings in the case of people with darker skin pigmentations [1]. This problem, which was first raised 32 years ago, has not been solved until today. The above-mentioned panel of external FDA experts discussed all the factors that may affect the accuracy and performance of pulse oximeters, the available evidence about the accuracy of them, the recommendations for health care providers and patients and the type of data that should be provided by manufacturers to assess the accuracy of pulse oximeter as well as to guide other regulatory actions as needed. Once collected all the opinions and the recommendations of the experts, the FDA panel will provide information how to improve the assessment of pulse oximeters to ensure the best accuracy for all skin pigmentations, and whether pulse oximeters should be labeled with warnings about their limitations. The clinical findings of the past 3 years provide an obligation for developing and validating pulse oximeters without dependence on the subject’s skin melanosome; subjects with darker skin tones have significantly larger and more concentrated melanosomes that increase the absorption cross section of melanin resulting in enhanced light absorption.

Therefore, any medical equipment that leverages near-infrared light interacting with the skin should not show discordance in its performance across people with different skin tones.

We think the problem with pulse oximeters is also relevant for other type of noninvasive optical oximeters. The noninvasive estimation of cerebrovascular blood oxygen saturation (mostly by a sensor attached to the forehead) by near-infrared spectroscopy (NIRS)-based cerebral oximetry equipment has been increasingly used in many clinical areas such as (1) cardiac, vascular and thoracic surgery for detecting cerebral hypoxia–ischemia as well as developing interventions to avoid and improve hypoxic brain injury and (2) intensive care for monitoring and modifying brain oxygenation in patients at risk of hypoxic ischemic brain injury after cardiac arrest [2] as well as for monitoring the early effects of ventilatory rescue therapies on brain oxygenation in mechanically ventilated COVID-19 patients with acute respiratory distress syndrome [3]. NIRS-based cerebral oximeters, first described more than 40 years ago, measure cerebrovascular oxygen saturation (StO_2_) of the hemoglobin present primarily in small vessels (< 1 mm in diameter) such as the capillary, arteriolar and venular bed of the cerebral cortex. Already in 2005, Wassenaar et al. [4] highlighted that, in patients with a dark pigmented skin, StO_2_ should be interpreted with caution, as melanin clearly interferes with the quality of the reflected NIRS signal. In modern cerebral oximeters, wavelengths have been added to enable a more precise determination of hemoglobin and to be less sensitive to skin pigmentation. Very recently, the robustness of two commercial NIRS-based cerebral oximeters (using four or five wavelengths) to variations in skin pigmentation was evaluated using a tissue-simulating phantom; unexpectedly increasing melanin content decreased saturation values displayed by both devices [5]. The effect of skin pigmentation was particularly inflicted when using a neonatal sensor. Besides the skin pigmentation, ethnic/racial differences in skin anatomy should be considered too as an additional cause of bias in optical oximetry.

Considering the extensive clinical use of cerebral oximetry, we strongly suggest that the FDA and the other regulatory bodies should deliberate also how to improve NIRS-based cerebral oximeters to best ensure accuracy for all skin pigmentations and eventually recommend labeling them with warnings about their limitations. Furthermore, we urge manufacturers producing optical cerebral oximeters to validate their devices and sensors regarding their robustness against skin pigmentations of different strength.

## Data Availability

Not applicable.

## References

[1] Shi C, Goodall M, Dumville J, Hill J, Norman G, Hamer O (2022). The accuracy of pulse oximetry in measuring oxygen saturation by levels of skin pigmentation: a systematic review and meta-analysis. BMC Med.

[2] Skrifvars MB, Sekhon M, Åneman EA (2021). Monitoring and modifying brain oxygenation in patients at risk of hypoxic ischaemic brain injury after cardiac arrest. Crit Care.

[3] Robba C, Ball L, Battaglini D, Cardim D, Moncalvo E, Brunetti I (2021). Early effects of ventilatory rescue therapies on systemic and cerebral oxygenation in mechanically ventilated COVID-19 patients with acute respiratory distress syndrome: a prospective observational study. Crit Care.

[4] Wassenaar EB, Van den Brand JG (2005). Reliability of near-infrared spectroscopy in people with dark skin pigmentation. J Clin Monit Comput.

[5] Afshari A, Saager RB, Burgos D, Vogt WC, Wang J, Mendoza G (2022). Evaluation of the robustness of cerebral oximetry to variations in skin pigmentation using a tissue-simulating phantom. Biomed Opt Express.

